# Improving Education in Medical Statistics: Implementing a Blended Learning Model in the Existing Curriculum

**DOI:** 10.1371/journal.pone.0148882

**Published:** 2016-02-09

**Authors:** Natasa M. Milic, Goran Z. Trajkovic, Zoran M. Bukumiric, Andja Cirkovic, Ivan M. Nikolic, Jelena S. Milin, Nikola V. Milic, Marko D. Savic, Aleksandar M. Corac, Jelena M. Marinkovic, Dejana M. Stanisavljevic

**Affiliations:** 1 Institute for Medical Statistics and Informatics, Faculty of Medicine, University of Belgrade, Belgrade, Serbia; 2 Department of Public Health, Medical Faculty, University of Pristina, Kosovska Mitrovica, Serbia; Universidad Europea de Madrid, SPAIN

## Abstract

**Background:**

Although recent studies report on the benefits of blended learning in improving medical student education, there is still no empirical evidence on the relative effectiveness of blended over traditional learning approaches in medical statistics. We implemented blended along with on-site (i.e. face-to-face) learning to further assess the potential value of web-based learning in medical statistics.

**Methods:**

This was a prospective study conducted with third year medical undergraduate students attending the Faculty of Medicine, University of Belgrade, who passed (440 of 545) the final exam of the obligatory introductory statistics course during 2013–14. Student statistics achievements were stratified based on the two methods of education delivery: blended learning and on-site learning. Blended learning included a combination of face-to-face and distance learning methodologies integrated into a single course.

**Results:**

Mean exam scores for the blended learning student group were higher than for the on-site student group for both final statistics score (89.36±6.60 vs. 86.06±8.48; p = 0.001) and knowledge test score (7.88±1.30 vs. 7.51±1.36; p = 0.023) with a medium effect size. There were no differences in sex or study duration between the groups. Current grade point average (GPA) was higher in the blended group. In a multivariable regression model, current GPA and knowledge test scores were associated with the final statistics score after adjusting for study duration and learning modality (p<0.001).

**Conclusion:**

This study provides empirical evidence to support educator decisions to implement different learning environments for teaching medical statistics to undergraduate medical students. Blended and on-site training formats led to similar knowledge acquisition; however, students with higher GPA preferred the technology assisted learning format. Implementation of blended learning approaches can be considered an attractive, cost-effective, and efficient alternative to traditional classroom training in medical statistics.

## Background

There are ongoing changes in the utilization of modern information and communication technologies in medical education [1–4]. An increasing number of students are switching from traditional to online classrooms [5]. Research has suggested that well organized online classrooms are as effective and of comparable quality as traditional classrooms [6–8]. The main advantage of web-based education is its flexibility, allowing students to access content from diverse locations, at a convenient time. It encourages learning self-management, with the ability to exchange links to related information [9–12].

Online and face-to-face traditional instruction formats have their own respective strengths and weaknesses. Neither is better, but rather they are complementary. This confirms the benefit of what is known as “blended learning”, which includes a combination of face-to-face traditional and distance (online) learning methodologies integrated into a single course [13,14]. Integrating “blended learning” as part of the educational curriculum can provide teachers with a broad spectrum of tools to create and deliver effective quality education [15].

A recently published systematic review of original research articles from respected physiology journals (n = 703) indicated that educators urgently need to improve training in data analysis and research data presentation [16]. The paper presented evidence that the “one size fits all” approach in relaying medical statistics knowledge does not necessarily result in a proper understanding of statistical methods in different research areas. Consistent with this evidence, an online learning platform was developed in the Department for Medical Statistics and Informatics, Faculty of Medicine, University of Belgrade to improve knowledge acquisition. Although several studies report the benefits of using blended learning [17,18], there is still little empirical evidence as to its relative effectiveness compared to traditional learning approaches in medical statistics. We performed a study implementing blended along with on-site (i.e. traditional) learning to further assess the potential value of web-based learning in medical statistics. As our previous research has indicated that younger age, positive self–rated ability in mathematics, and higher current grade point average (GPA) might be related to better knowledge acquisition during medical statistics courses [19], a second aim of this study was to explore the relationship between student characteristics and final statistics achievement.

## Method

This was a prospective trial conducted with third year medical undergraduate students attending the Faculty of Medicine, University of Belgrade, who passed (440 of 545) the final exam of the obligatory introductory medical statistics course taught 2013–14. Two methods of education delivery were tested: blended learning and traditional on-site. Students were asked to choose their learning method at the beginning of their statistics course.

The content of the introductory medical statistics course was developed using established principles of curriculum development. It included: 10 lectures, 20 hours of practical class work and eight hours of seminars, that covered statistical analysis and study design, including types of data, descriptive statistics, normal distribution, sampling distribution, central limit theorem, confidence intervals, hypothesis testing for continuous and categorical data, simple linear regression and measures of association, applying various statistical software.

Learning objectives, course materials and course lecture slides were identical for the blended and on-site formats of the course, and were taught by the same instructors (Table 1). The blended teaching approach was supported by the multimedia didactic materials which the students studied by computer via the Internet, using the University’s Moodle 2.5 platform. These materials contained the same information as that used in the face-to-face classes. Students were provided with reading material to accompany or complement each lecture, as well as copies of all lecture slides. Both online and on-site courses included structured live group activities and case discussions, in addition to the formal lectures. Online students also had the option of posting questions through a web portal to facilitate discussion with fellow students and course faculty. On-site courses included time for questions and discussion during and after lectures.

**Table 1:** The elements of the traditional and blended learning methods in medical statistics.

Element	Traditional course (3 h of classroom meeting each week)	Blended-learning course (1 h of classroom meeting each week)
Delivery of material	Lectures supported with PowerPoint presentation	Web-site, online materials
Interaction with materials	Text books, notes, homework, quizzes, classroom activities	Multimedia, web browsing, homework, quizzes, classroom activities
Interaction with the teacher	Classroom discussion, face to face questions, consultation	Web announcements, forum, face to face questions, consultation
Interaction among students	Group work, classroom discussions, projects	Web forum, e-mail, group work, classroom discussions, projects
Intra-action	Classroom discussions, group work	Classroom discussions, group work, web forum
Knowledge testing	Knowledge test, final exam	Knowledge test, final exam

The formal evaluation of student achievement was identical for both learning modalities and consisted of:

1. Course activities throughout the year
2. A written knowledge test: Administered on the last day of the statistics course and written by the course instructors. It consisted of 20 multiple-choice items, each with four response alternatives. Test scores ranged from 0–10. Questions related to descriptive statistics (frequency tables, measures of central tendency, measures of dispersion, types of distributions, and measures of association) and inferential statistics (basic concepts in inferential statistics, estimation, hypothesis testing). Students were given 40 minutes to complete the knowledge test. If a student failed the test, or otherwise was unable to participate, a new knowledge test had to be taken within seven days. Only the second test results were used for this group of students.
3. Final exam: consisted of a problem-based written portion and an oral examination, both of which could be taken by the student 3–12 weeks after course completion. The final score for overall statistics achievement was calculated by summing the activities during classes (weighted 0.2) and the score of the knowledge test (weighted 0.1), problem-based written exam (weighted 0.2), and oral examination (weighted 0.5). Course instructors involved in the oral examination were blinded to the student’s choice of learning method.

The outcome variables in this study were student statistics achievements measured in four ways. The final statistics score (ranging from 0 to 100) was defined as the primary endpoint. The knowledge test score (0–10), passing the knowledge test upon the first attempt during the last class day, and passing the final exam upon first attempt at the earliest available date 3 weeks after course completion were defined as secondary endpoints. All four outcome variables were compared between the two learning groups to assess the relative efficacy of the blended learning approach. Given the non-equivalent control group design, the primary threats to the validity of this testing were the differences in student characteristics between the two groups. For that reason, prior to the analyses of the learning outcomes, we examined the equivalence of the students based on prior academic performance (GPA ranging from 0 to 10), study duration (within vs. outside of scheduled time frame), and gender (independent variables).

Ethical approval for the study was obtained from the Institutional Review Board (IRB) of the Faculty of Medicine, University of Belgrade. The purpose of the study was explained to the students and their oral consents were obtained and documented in their records at the beginning of the introductory biostatistics course. The IRB approved the use of oral consent as there was no potential harm to the study participants.

### Statistical Analysis

Descriptive statistics were calculated for the baseline student characteristics and outcome measures, knowledge test and final scores. Baseline differences between groups were analyzed using Students t-test for continuous variables, and the Pearson chi-squared test for categorical variables. Second, we assessed the relative size of the effect based on standardized estimates of effect size according to Cohen’s benchmarks [20], which defined *d* as the difference between the means divided by pooled SD. Sensitivity analysis was performed to explore how the variable GPA is related to learning method by dividing the sample according to GPA (high ≥ 8 and low < 8). The univariable and multivariable linear regression analyses were used to determine factors related to student statistics achievement measured by the knowledge test and final statistics scores, adjusted based on prior academic performance, study duration, and gender. The univariable and multivariable logistic regressions were used to determine the independent predictors for passing the final exam and knowledge test on the first attempt, adjusted for prior academic performance, study duration, and gender. Adjustment variables were included in the multivariable regression analysis if they were significant at the p < 0.1 level according to the results of the univariable analysis. Results were expressed as linear regression coefficients (B) or the odds ratios (OR) where appropriate, and their 95% confidence intervals (CI). All tests were two-tailed. P < 0.05 was considered statistically significant. The achieved statistical power for the study was 0.948, with *a =* 0.05 for a medium effect size (*d =* 0.43) based on an independent *t*-test. The IBM SPSS 21 (Chicago, IL, 2012) package was used for these analyses.

## Results

Four hundred forty of 545 medical students passed the final statistics exam during the 2013–14 school year. Most participants were female (67%), 79% of the students enrolled in medical statistics course had completed their scheduled course work within the expected time frame, and their GPA was 8.21±1.00. Eighty seven students (19.8%) used the blended method. The average final statistics score was 88.72±8.24, and the average knowledge test score was 7.58±1.35. The majority of students passed the final statistics exam upon first attempt at the earliest available date (63.2%), as did the majority (88.6%) upon first attempt for the knowledge test. Descriptive statistics of student characteristics and outcome measures for all students, and according to the groups (blended vs. on-site) are presented in Table 2. There were no statistically significant differences in sex and study duration between the groups. The current GPA was significantly higher in the blended group. As can be seen in Table 2, exam scores for the blended learning group were statistically significantly higher than for the on-site group for both final statistics and knowledge test scores. The calculated effect sizes for the final score and knowledge test difference were 0.43 and 0.28, respectively, which stands for a “medium” effect size according to Cohen’s guidelines for describing effect sizes. The dropout rates (i.e. students who did not attempt to pass the final exam during the school year) were 21.4% and 9.4% for the on-site and blended groups, respectively, and indicate that students from the blended learning group were more likely to complete their course obligations in a timely manner. However, there were no statistically significant differences between the groups in passing the final exam upon first attempt at the earliest available date and knowledge test on the first attempt. As the current GPA was higher in the blended group, we tested whether students with high GPA differed from those with low GPA in a sensitivity analysis. There was a difference in the mean final scores for students with a low GPA between the on-site and blended learning groups (81.31±8.46 vs. 85.50±6.18, p = 0.030), but not for students with a high GPA (89.87±6.32 vs. 90.58±6.29, p = 0.428).

**Table 2:** Descriptive statistics of students characteristics and learning outcomes.

Variables	Total	Blended	On-site	Effect size	p value^#^
**Students characteristics**					
Female, n (%)	295 (67%)	59 (68%)	236 (67%)		0.864
Studying within time frame, n (%)	346 (79%)	72 (83%)	274 (78%)		0.405
GPA, mean±SD	8.21±1.00	8.60±0.98	8.11±0.98		**<0.001**
**Outcome variables**					
Knowledge test score (0–10), mean±SD	7.58±1.35	7.88±1.30	7.51±1.36	0.28	**0.023**
First attempt, n (%)	390 (89%)	82 (94%)	308 (87%)		0.065
Final statistics score (0–100), mean±SD	88.72±8.24	89.36±6.60	86.06±8.48	0.43	**0.001**
Earliest available date, n (%)	278 (63%)	60 (69%)	218 (62%)		0.212
Have not tried to pass final exam, n (%)	105 (19.3%)	9 (9.4%)	96 (21.4%)		0.007

GPA–Grade point average; SD–standard deviation

^#^—according to chi-square or t-test where appropriate

In the univariable linear regression analysis, the variables of the learning method, study duration, current GPA, and knowledge test scores were significantly associated with final statistics scores. In a multivariable regression model, only current GPA and knowledge test scores were significantly associated with the final score, after adjusting for study duration and learning modality. Students with a higher current GPA and better knowledge test scores had higher final scores than those with poor current GPA and lower knowledge test scores (Table 3).

**Table 3:** Variables associated with student statistics achievement–final statistics score.

Variables	Univariable		Multivariable	
**Final score**	**B (95% CI)**	**p value**	**B (95%CI)**	**p value**
Learning method	0.41 (0.18–0.64)	**<0.001**	0.15 (-0.04–0.33)	0.124
Female	0.03 (-0.17–0.23)	0.774		
Study duration	0.86 (0.64–1.08)	**<0.001**	0.08 (-0.15–0.30)	0.499
GPA	0.59 (0.51–0.66)	**<0.001**	0.39 (0.28–0.50)	**<0.001**
Knowledge test	0.38 (0.32–0.44)	**<0.001**	0.20 (0.14–0.27)	**<0.001**
**First available date**	**OR (95%CI)**	**p value**	**OR (95%CI)**	**p value**
Learning method	1.38 (0.83–2.27)	0.213		
Female	1.03 (0.68–1.55)	0.897		
Study duration	3.10 (1.92–5.00)	**<0.001**	1.07 (0.59–1.96)	0.816
GPA	2.37 (1.88–2.98)	**<0.001**	1.82 (1.33–2.50)	**<0.001**
Knowledge test	1.72 (1.46–2.02)	**<0.001**	1.35 (1.11–1.65)	**0.003**

GPA–Grade point average; B–Standardized linear regression coefficient; CI–Confidence interval; OR–Odds ratio

According to the results of the univariable analysis, factors associated with passing the final statistics exam upon first attempt at the earliest available date were study duration, current GPA and knowledge test score. In a multivariable logistic regression model, passing the final statistics exam upon first attempt at the earliest available date was positively related with GPA and knowledge test score, after adjusting for study duration (Table 3).

Learning method, study duration, and current GPA were significantly associated with the knowledge test score. In a multivariable regression model, only current GPA was significantly associated with the knowledge test score, after adjusting for study duration and learning method. Students with higher current GPA had higher knowledge test scores than those with lower current GPA. Factors associated with passing the knowledge test during the first attempt were study duration and current GPA. In a multivariable logistic regression model, passing the knowledge test during the first attempt was positively related only to GPA, after adjusting for study duration and learning modality (Table 4).

**Table 4:** Variables associated with student statistics achievement–knowledge test.

Variables	Univariable		Multivariable	
**Knowledge test**	**B (95%CI)**	**p value**	**B (95%CI)**	**p value**
Learning method	0.26 (0.04–0.48)	**0.021**	-0.037 (-0.22–0.14)	0.689
Female	-0.06 (-0.25–1.13)	0.541		
Study duration	0.78 (0.57–0.98)	**<0.001**	-0.06 (-0.27–0.16)	0.603
GPA	0.56 (0.49–0.63)	**<0.001**	0.59 (0.50–0.68)	**<0.001**
**First attempt**	**OR (95%CI)**	**p value**	**OR (95%CI)**	**p value**
Learning method	2.40 (0.92–6.23)	0.073	1.62 (0.59–4.46)	0.346
Female	1.28 (0.70–2.36)	0.421		
Study duration	4.74 (2.55–8.82)	**<0.001**	1.39 (0.63–3.08)	0.410
GPA	3.01 (2.08–4.35)	**<0.001**	2.79 (1.75–4.45)	**<0.001**

GPA–Grade point average; B–Standardized linear regression coefficient; CI–Confidence interval; OR–Odds ratio

## Discussion

This study provides further evidence supporting the effectiveness of a blended learning format for biostatistics classes for undergraduate medical students. Overall, student performance was higher in those using blended learning than in the traditional on-site training group with a medium effect size. A knowledge gain favoring the blended learning model was detected for the final statistics and knowledge test scores. An important finding was that students with higher GPA scores more often chose an online classroom learning environment, indicating a preference for learning formats that are more information and communication technology oriented. It also was demonstrated in our student population, that GPA and knowledge test scores were associated with final statistics scores, after adjusting for study duration and learning method, as potential confounding factors.

Online learning is strongly reliant on user “satisfaction and knowledge” [8], giving the student educational expertise comparable only to that acquired in small study groups in traditional settings [21]. It is suggested that the advantages of online learning include its simplicity, its flexibility in fitting the needs of the user, reducing the problem of time management, with less anxiety and high problem solving efficacy. Having the flexibility to fit user preference is probably the biggest advantage of online classroom, but its effectiveness lies in its backbone:—The asynchronous ability to exchange information, cost-saving, personalized learning, increased accessibility, ease of distribution and updating content are just some examples of the advantages possessed by the online classrooms. In a study carried out by Hui et al. [22], the authors demonstrated that technology based learning improves student knowledge acquisition which requires abstract conceptualization and reflective observation, but adversely affects student ability to obtain knowledge requiring concrete experience. McGready et al. demonstrated that an introductory biostatistics course can be successfully delivered online based on the findings that student outcomes were comparable to those of an on-site course [23].

Although online learning has clearly displayed advantages compared to its traditional counterpart [24,25], no clear evidence has existed until recently to indicate whether blended or strictly online courses are better. Some authors have reported no difference in outcomes between these models of knowledge delivery [26–29]. Lim et al., in an empirical investigation of student achievement using different learning styles, showed that students enrolled in online and blended courses had significantly higher achievement rates than their traditional colleagues, but no significant differences were found between the blended and online groups [30]. So and Brush were investigating the influence of collaboration with faculty and other students on course performance, and discovered that students who were exposed to a more collaborative environment tended to be more satisfied with their distance course than those who perceived lesser levels of exposure to collaborative learning [31]. Kiviniemi demonstrated that well implemented blended learning models may have strong potential for improving student learning outcomes in health sciences studies [32]. Similarly, a study by Delilaoglu found that the students had similar levels of achievement, after adjusting for pre-test and GPA scores, which is consistent with our results [33]. The research done by Larson reported no significant differences among the three education delivery methods (on-site, online and blended learning) as measured by exam scores and final grades. Consequently, they concluded that online and blended methods are at least equivalent to the face-to-face method, but no data were reported about previous GPA [26]. A rare study which compared traditional and blended class types in the field of statistics directly demonstrated the conclusion that there is no significant influence of gender, ethnicity, age or class type, but there is a significant influence of student incoming GRE Quantitative scores on student performance [34].

A comprehensive meta-analysis recently conducted by Teachers College, Columbia University [5], indicated that students studying in online classrooms had moderately better performances than those receiving instruction in traditional classrooms, which is similar to the effect size from our study. The difference was significant when comparing blended learning to that of the traditional classroom, although no major differences were found when comparing it to the purely online classroom. The important finding of this study which cannot be ignored is that there is confounding of the results with respect to involving more learning time, additional instructional resources, as well as course elements that encourage interactions among learners for blended learning format. This finding leaves open the possibility to study these and other additional practice variables that may contribute to the positive outcomes for blended learning, and necessitates further research into the development of different blending formats for different types of learners.

Statistics is often portrayed as the most difficult, anxiety-provoking, and a critical subject for the average medical student. It, therefore, is expected to be the most revolutionary in its efforts to improve the learning environment [35,36]. Blended courses appear optimal to facilitate learning statistics, and are comparable to their traditional counterparts. Students are still left with the choice of the learning model which will suit them best, whether traditional or blended. Data suggest that students with higher GPA scores tend to choose blended learning. This could be explained by the fact that blended learning provides maximum productivity with minimum wasted time in the overall very time-consuming studies of medicine. However, the implementation of hybrid educational methods can be demanding on the teaching staff, especially when it comes to organization and the clarity of course requirements [37]. Materials should be designed to increase motivation, meet student expectations, and above all highlight the subject’s key points. Lastly, the educators must be capable of anticipating problems that arise during this interactive educational activity and be able to develop strategies for their resolution. The demonstrated diverse capabilities of web-based technologies support the development and implementation of blended learning curricula in various educational settings, one of which may be medical statistics.

### Study limitations

Study results should be interpreted taking into account our study limitations. Our study is a single center experience and the study design was not truly experimental. Future research is needed to examine other important outcomes, such as knowledge retention, student satisfaction and its effect on educators with respect to attitudes and effort involved.

## Conclusion

This study provides empirical evidence to provide different learning environments for teaching medical statistics to undergraduate medical students. Blended and on-site training formats in medical statistics led to similar knowledge acquisition. Factors associated with medical student statistics learning outcomes are GPA and knowledge test score. Implementation of blended learning approaches can be considered as an attractive, cost-effective, and efficient alternative to on-site classroom training in medical statistics.
